# Nailing it: Investigation of elephant toenails for retrospective analysis of adrenal and reproductive hormones

**DOI:** 10.1093/conphys/coae048

**Published:** 2024-08-02

**Authors:** Garrett Rich, Rebecca Stennett, Marie Galloway, Mike McClure, Rebecca Riley, Elizabeth W Freeman, Kathleen E Hunt

**Affiliations:** Department of Biology, George Mason University, 4400 University Drive, Fairfax, VA 22030, USA; The Maryland Zoo in Baltimore, 1 Safari Place Baltimore, MD 21217, USA; Smithsonian’s National Zoo and Conservation Biology Institute, 3001 Connecticut Ave NW, Washington, DC 20008, USA; The Maryland Zoo in Baltimore, 1 Safari Place Baltimore, MD 21217, USA; Smithsonian’s National Zoo and Conservation Biology Institute, 3001 Connecticut Ave NW, Washington, DC 20008, USA; School of Integrative Studies, George Mason University, 4400 University Drive, Fairfax, VA 22030, USA; Department of Biology, George Mason University, 4400 University Drive, Fairfax, VA 22030, USA; Smithsonian-Mason School of Conservation, 1500 Remount Road, Front Royal, VA 22630, USA

**Keywords:** Elephants, hormones, keratin, noninvasive, reproduction, stress

## Abstract

Hormone monitoring of at-risk species can be valuable for evaluation of individual physiological status. Traditional non-invasive endocrine monitoring from urine and faeces typically captures only a short window in time, poorly reflecting long-term hormone fluctuations. We examined toenail trimmings collected from African (*Loxodonta africana*) and Asian (*Elephas maximus*) elephants during routine foot care, to determine if long-term hormone patterns are preserved in these slow-growing keratinized tissues. We first measured the growth rate of elephant toenails biweekly for one year, to establish the temporal delay between deposition of hormones into nail tissue (at the proximal nail bed) and collection of toenail trimmings months later (at the distal tip of the nail). In African elephants, toenails grew ~0.18 ± 0.015 mm/day (mean ± SEM) and in Asian elephants, toenails grew ~0.24 ± 0.034 mm/day. This slow growth rate, combined with the large toenail size of elephants, may mean that toenails could contain a ‘hormone timeline’ of over a year between the nail bed and nail tip. Progesterone, testosterone and cortisol were readily detectable using commercial enzyme immunoassays, and all assays passed validations, indicating that these hormones can be accurately quantified in elephant toenail extract. In most cases, variations in hormone concentrations reflected expected physiological patterns for adult females and males (e.g. ovarian cycling and musth) and matched individual health records from participating zoos. Progesterone patterns aligned with our calculations of temporal delay, aligning with female ovarian cycling from over six months prior. Unexpectedly, male testosterone patterns aligned with current musth status at the time of sample collection (i.e. rather than prior musth status). Though this sample type will require further study, these results indicate that preserved hormone patterns in elephant toenails could give conservationists a new tool to aid management of elephant populations.

## Teaser

We investigated the use of toenails for retrospective, non-invasive analysis of progesterone, testosterone, and cortisol in African and Asian elephants, and measured toenail growth rate across one year. All hormones were detectable in toenail extract, and hormonal variations in different trimmings of toenail reflected expected individual variation in endocrine status.

## Introduction

Endocrinology techniques are a valuable tool for the conservation and management of wildlife. Evaluation of hormonal states can provide researchers with critical information about an individual’s reproductive physiology and responses to stress. In mammals, the steroid hormones progesterone and testosterone often reflect reproductive states including pregnancy, sexual maturity and reproductive cycles (Lasley and Kirkpatrick, 1991; McCormick and Romero, 2017; Melica *et al.,* 2021), while the glucocorticoids (cortisol, corticosterone) provide insight into exposure to stressors and the resulting impacts on health (Sheriff *et al.,* 2011; Matas *et al.,* 2016). Evaluating hormonal states of *in situ* and *ex situ* wildlife populations can be accomplished through a wide variety of sample matrices. Traditional endocrine sampling relies on collection of serum or plasma, but alternative sample types such as faeces and urine are increasingly used due to the potential for minimally invasive or non-invasive collection from living individuals in captivity and the wild (Lasley and Kirkpatrick, 1991; Sheriff *et al.,* 2011; Heimbürge *et al.,* 2019; Palme, 2019).

Elephant physiology is marked by notably long-term hormonal changes corresponding with reproductive and potentially stressful events. In female elephants, the 3-month-long ovarian cycle is characterized by a prolonged elevation of progestagens during a 10-week luteal phase (Hess *et al.,* 1983; Plotka *et al.,* 1988). Pregnancy in elephants is characterized by elevated progestagens continuing after the luteal phase and throughout the 22-month gestation period (Hess *et al.,* 1983; Hodges, 1998). In male elephants, the unique male reproductive state of musth can be detected via a significant elevation in circulating androgens (testosterone, etc.) that can last weeks to months. Musth has an unpredictable onset in different individuals and involves changes in behavior that can pose challenges for management of male elephants *ex situ* (Poole *et al.,* 1984; Lincoln and Ratnasooriya, 1996; Brown, 2000; LaDue *et al.,* 2021). Glucocorticoids, which can become detrimental to health if elevated over extended periods of time (Moberg, 2000; Sheriff *et al.,* 2011), are often monitored as a tool for management of elephant populations (Ahlering *et al.,* 2011; Fanson *et al.,* 2013; Brown *et al.,* 2019). Although monitoring hormones can, in theory, facilitate management decisions, collection of samples from elephants is often challenging. Blood collection *ex situ* requires training for minimally invasive collection and from *in situ* individuals is logistically difficult if not dangerous. Urine and faecal samples can be challenging to match to the individual, and repeated samples across months are not always available, particularly *in situ*, which challenges ability to monitor the prolonged (multi-month) reproductive cycles of elephants.

A potential alternative endocrine matrix for evaluation is the keratin tissues (e.g. hair, feathers, scales, nails), which may enable retrospective evaluation of endocrine events that occurred in prior months or years (Matas *et al.,* 2016; Kalliokoski *et al.,* 2019). All vertebrate keratin tissues studied to date have been shown to accumulate steroid hormones as they grow. Some of these tissues grow continuously in a linear fashion, extending distally from a well-vascularized epidermal growth zone (e.g. hair follicle, nail bed), and these tissues can represent an endocrine ‘time series’ with points along the keratin structure containing hormones that were deposited at different times. Thus, an entire sample can be used to reconstruct a detailed individual endocrine history that spans the time period of tissue growth (e.g. whale baleen, Hunt *et al.,* 2014, 2017b; human fingernail, Izawa *et al.,* 2021; seal vibrissae, Keogh *et al.,* 2021). Further, keratin samples can often be collected non-invasively (e.g. shed hair) or with minimal invasiveness (e.g. clipping of distal parts of hair or nail samples). The dry matrix of keratin has also been shown to preserve steroid hormones for decades even at room temperature (e.g. Hunt *et al.,* 2017a; Beattie and Romero, 2023). Long-term preservation of hormones could thus enable historic keratin samples from natural history museums to be used to reconstruct endocrine patterns of past populations, enabling comparison to present-day populations and enhancing ability to understand the effects of anthropogenic impacts (Koren *et al.,* 2002).

In elephants, keratin tissues such as toenails and tail hairs could allow evaluation of prolonged (multi-month) patterns of hormones, while providing a much broader timeframe than serum, faecal or urine sampling. While elephant tail hair has been investigated as a potential endocrine sample type for elephants, specifically for cortisol (Pokharel *et al.,* 2021), to our knowledge, no studies have evaluated elephant toenails as a potential sample matrix for any hormones. In *ex situ* populations, toenail trimmings are frequently removed from the distal part of the nail during routine foot care. Trimmings are usually discarded but could be used to retrospectively evaluate the endocrine status of the elephant. Natural history museums, as well, contain historic specimens of toenails that could be studied to evaluate endocrine patterns of past populations. Nails of humans (Palmeri *et al.,* 2000; Warnock *et al.,* 2010; Izawa *et al.,* 2015; Fischer *et al.,* 2020) and claws of other mammalian and non-mammalian species (turtles, Baxter-Gilbert *et al.,* 2014; seals, Karpovich *et al.,* 2020, Crain *et al.,* 2021; wolves, Roffler *et al.,* 2022) have proven to be informative endocrine sample types. However, each species’ unique nail or claw growth rate must be evaluated alongside endocrine evaluations in order to estimate the date of growth for the distal trimmed piece.

The overarching goal of our study was to assess the utility of African and Asian elephant toenails as a non-invasive hormone matrix. We hypothesized that the steroid hormones progesterone, testosterone and cortisol would be detectable in elephant toenail extract and that patterns in hormones across successively collected toenail trimmings would reflect the elephant’s endocrine status from the prior months or years, with a temporal lag determined by nail growth rate and length of the nail. Specific goals were to (i) measure the growth rate of elephant toenail in African and Asian elephants of both sexes, across a full year; (ii) determine whether progesterone, testosterone and cortisol are present and detectable in elephant toenail extract; (iii) validate (parallelism, accuracy) commercial enzyme immunoassay kits to quantify hormones in elephant toenail matrix; and (iv) perform preliminary biological validations by comparing patterns in toenail hormones to documented physiological status from keeper records.

## Materials and methods

### Sample collection

Toenail trimmings were collected monthly from two female and one male African elephants (*Loxodonta africana*) at the Maryland Zoo in Baltimore (September 2020 to August 2021; Table 1) and from three female and one male Asian elephants (*Elephas maximus*) at the Smithsonian’s National Zoo and Conservation Biology Institute (November 2020 to December 2021; Table 1). All elephants were well habituated to routine foot care by animal care staff, voluntarily allowed their feet to be handled, received positive reinforcement via food rewards and were not anesthetized or restrained for toenail trimming. Toenails were collected from front or hind feet based on the management practices of both zoos; specifically, African elephant toenail samples were collected from the hind feet and Asian elephant samples from the front feet. Upon collection, toenail trimmings were stored at −20°C for up to 6 months until transfer to George Mason University for analysis. Both participating zoos are accredited by the Association of Zoos and Aquariums and follow all recommended best practices for housing and husbandry of elephants in human care. This study followed all applicable local, state and federal regulations and was approved by both participating zoos and by the Institutional Animal Care and Use Committee of George Mason University.

**Table 1:** Details of individual elephants sampled in this study. Toenail trimmings were sampled from African (*n =* 3) and Asian (*n =* 4) elephants once a month. Maryland Zoo = The Maryland Zoo in Baltimore; NZCBI = the Smithsonian’s National Zoo and Conservation Biology Institute.

**Individual**	**Species**	**Zoo**	**SSP #**	**Sex**	**Age**	**Number of samples** ^**a**^
Anna^b^	*Loxodonta africana*	Maryland Zoo	138	Female	47	9
Felix^c^	*L. africana*	Maryland Zoo	339	Female	39	10
Samson	*L. africana*	Maryland Zoo	561	Male	14	10
Kamala^c^	*Elephas maximus*	NZCBI	145	Female	47	12
Maharani^c^	*E. maximus*	NZCBI	307	Female	32	14
Spike	*E. maximus*	NZCBI	141	Male	41	15
Swarna^b^	*E. maximus*	NZCBI	146	Female	47	13

a The number of toenails from that individual that were assayed for hormone content, i.e. excluding any samples that were collected but not assayed, such as same-day replicates (i.e. multiple toenail trimmings collected from the same day), samples with very low mass < 10 mg (known to result in inaccurate hormone data for dry sample types; ‘small sample effect’ (Hayward *et al.,* 2010, Berk *et al.,* 2016, Fernández Ajó *et al.,* 2022), or samples with intractable shape for pulverization.

b Irregular/acyclic female.

c Cyclic female.

Animal care staff routinely keep a log of reproductive events (e.g. indications of ovarian cycling or musth) and note any potentially stressful events. As not all female elephants experience regular ovarian cycling (Brown, 2000; Freeman *et al.,* 2009; Brown, 2019), females in this study were classed as either ‘cycling’ (i.e. experienced regular cycles of predictable occurrence and duration) or ‘irregular/acyclic’ (i.e. irregular cycles of unpredictable occurrence, or acyclic), based on keeper records and veterinary records of serum progesterone (Table 1). Finally, both males experienced one musth episode during the sample collection period, based on indicators of urine dribbling and temporal gland secretions, behavior, and/or endocrine status from faeces (LaDue *et al.,* 2022). These individual records were used for biological validations, e.g. comparison of known or inferred endocrine status (as described above) to patterns of hormone concentrations observed in toenail samples.

### Determination of toenail growth rate

**Fig. 1:**
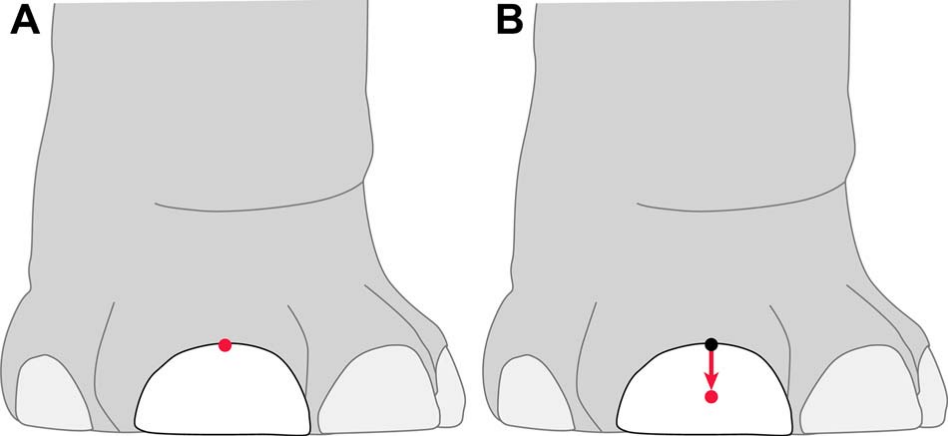
General methodology for determining growth rate of elephant toenails. (A) An initial marking was made near the cuticle of the elephant toenail at the start of the study date (indicated in red). (B) Every 2 weeks, the distance the mark travelled from the cuticle was measured (indicated by the red arrow). Growth rate was determined by dividing the distance travelled by the time interval between the initial mark and the new position. Image credit: Chase LaDue.

The toenail growth rate was determined by marking a shallow (~1 mm deep) horizontal groove on the surface near the cuticle of one toenail of each elephant. This groove did not penetrate through the dead outer layer of the nail and was similar to (but visually distinct from) surface markings that normally occur on elephant toenails during normal activity. Approximately every 2 weeks, the distance of the groove from the cuticle was photographed and measured to the nearest millimetre with a tape measure. Growth rate was evaluated in two ways: (i) biweekly growth rate, calculated for each 2-week period separately, as the distance between each new measurement and the prior measurement, divided by elapsed number of days (Fig. 1) and (ii) total growth rate across the entire study, calculated as the distance of groove from original measurement at the end of the study, divided by total number of days of the study period. All growth rates were converted to daily growth rates, millimetres per day, to standardize results.

### Toenail preparation and hormone extraction

Frozen toenail trimmings were transferred to George Mason University’s Sci-Tech Campus (Manassas, VA), stored at −80°C until drying and then air-dried in a fume hood. Samples were weighed daily starting on the seventh day of drying and considered dry when mass of the sample remained consistent (≤0.5 mg variation) across successive daily measurements. Dried toenail samples were then cleaned of any surface contamination with 70% isopropyl alcohol. Toenails were pulverized to a fine powder via a Dremel model 3000 (a powered rotary grinder) with flexible shaft attachment using a tungsten-carbide cylindrical tip. The Dremel, other tools and workspace were thoroughly cleaned with a 70% alcohol solution between samples to prevent cross-contamination. A Sartorius Entris II digital scale was used to weigh 10 ± 1 mg of toenail powder (recorded to the nearest 0.1 mg); this target mass was selected following published findings for minimum sample mass of other mammalian keratin samples (Hayward *et al.,* 2010; Berk *et al.,* 2016; Fernández Ajó *et al.,* 2022). During weighing of dried toenail powder, an Ohaus Ion 100A ionizer was placed next to the scale to reduce the effects of static charge on apparent sample mass. The weighed toenail powder was then transferred to a 16 × 100 mm borosilicate glass extraction tube, 4.00 ml of 100% methanol was added and tubes were vortexed for 1 h on a rack shaker (Glas-Col Large Capacity Mixer, at a speed setting of 40) and then centrifuged at 3000 rpm for 15 min (Sorvall ST4R centrifuge). Following centrifugation, 3.50 mL of the methanol extract (containing hormones) was pipetted into to a 13 × 100 mm borosilicate glass tube (final hormone data were corrected for percentage of supernatant that was not recovered), and the methanol was evaporated via a Thermo Scientific Savant SpeedVac rotary vacuum concentrator (model #SPD1030) at 45°C under vacuum until all samples were dry. Once dry, 500 μl of assay buffer (#X065; Arbor Assays, Ann Arbor, MI) was added to each tube, and the dried hormones were resuspended via vortexing for 1 min at medium speed on the rack shaker, sonication for 5 min (Branson Ultrasonic Bath M3800) and a final manual vortex for 20 sec at high speed on a Vortex Genie 2. The resulting extract was considered the ‘1:1’ or full-strength extract. Extracts were pipetted to O-ring-capped vaporproof cryovials for storage at −80°C until assayed within 6 months.

### Hormone assays

Hormone concentrations from toenail samples were determined via commercial enzyme immunoassay (EIA) kits for progesterone, testosterone and cortisol (catalog # K025, K032 and K003, respectively; Arbor Assays, Ann Arbor, MI; arborassays.com). Parallelism was assessed via comparison of the slopes of the binding curve of serially diluted extract pools (eight dilutions spanning 1:1–1:128) for each sex and each species to the slope of the hormone standards. An appropriate dilution for each hormone for each species was then selected based on the 50% binding point of the parallelism results, as follows: progesterone was assayed at a 1:4 dilution for both species; testosterone was assayed at 1:51 for African elephants and at 1:4 for Asian elephants; and cortisol was assayed at 1:4 for African elephants and 1:1 for Asian elephants. Dilutions were the same for males and females of the same species. Due to generally low cortisol content of elephant toenail extract, cortisol parallelism was assessed with a unique sample pool consisting specifically of high-apparent-cortisol samples from both sexes. That is, some samples were first assayed individually in order to identify those that had relatively high cortisol concentration, and then a ‘high-hormone pool’ was created using just those samples, thereby ensuring that the pool would have enough cortisol for parallelism assessment. Further, cortisol parallelism in Asian elephant also necessitated the use of a 1:5 dilution of assay antibody and conjugate to improve precision of the assay at very low concentrations; this 1:5 dilution has been comprehensively tested for low-cortisol samples in our lab and produces improved precision at high percent bounds while retaining acceptably low inter- and intra-assay variation (see below).

After successful parallelism validations, accuracy (matrix effect) validations were then performed to assess the ability of each assay to accurately distinguish high from low concentrations in the presence of sample matrix (toenail extract). Accuracy validations were performed at the aforementioned dilutions, by comparing hormone concentrations in a set of standards spiked with pooled diluted extract (equal volumes of standard and pool) to a standard curve spiked only with assay buffer (equal volumes of standard and buffer). Due to limited sample volume, assay accuracy was assessed for each hormone using pools from both males and females of the same species and not for each sex separately.

All assays followed the manufacturer’s protocols (available at www.arborassays.com), with two changes. First, one additional low-dose standard was added to each assay by extending the standard curve by one additional dilution, using the same process used to create the previous standards (for progesterone and cortisol assays, 250 μl buffer + 250 μl of previous standard; for the testosterone assay, 300 μl buffer + 200 μl of previous standard). Second, the cortisol assay was run in X065 buffer (rather than its usual X053 buffer) after consultation with the manufacturer, in order to streamline extraction methodology. Standards, pure hormone controls and samples were assayed in duplicate, with non-specific binding (‘NSB’) wells and zero-dose wells (‘maximum binding’) wells in quadruplicate. Each elephant’s samples were assayed in the same 96-well microplate with a full standard curve and control. Percentage binding of each sample (percentage of label bound) of each well was then used to interpolate the concentrations of the sample extracts with four-parameter logistic curve fits using Prism v.9 for OSX (www.graphpad.com). Cross-reactivities are reported in the manufacturer’s protocols (www.arborassays.com) and are further described in Hunt *et al.* (2017b). All assays were inspected for good fit of standard curve, normal NSBs, normal NSB/zero ratio, and coefficient of variation (%CV) of optical densities <10% for all standards and samples (i.e. to identify any cases of pipetting error; any sample with %CV >10% was re-assayed). Assay precision was assessed via calculation of %CV of interpolated hormone concentrations of African and Asian elephant toenail extract pools (i.e. one pool for each species) assayed in six different assays (inter-assay variation) and also assayed six times within one assay (intra-assay variation); all intra-assay %CVs were below 4% and all inter-assay %CVs were below 7% (see Table S1 in Supplementary Information for details). Any single anomalous standard was excluded from the standard curve; any assay with two or more anomalous standards was re-assayed. Final data were expressed in nanograms per gram of hormone per toenail powder.

### Statistical analyses

Toenail growth rate data were inspected for anomalous results. Four measurements that resulted in apparent negative growth rates were assumed to be due to measurement or data recording error and were subsequently removed from the dataset. An average of the biweekly growth rate for each individual was calculated for comparison to their total growth rate. Each individual was assigned an overall average growth rate by averaging that elephant’s biweekly average growth rate with that elephant’s total growth rate. Species growth rate was then estimated for African elephants and Asian elephants separately by averaging results for all individuals of that species (i.e. averaging the individual averages). Finally, African and Asian elephant growth rates were averaged to produce a single estimated toenail growth rate for Elephantidae. Differences in average growth rate between species were assessed via Welch’s *t*-tests.

Assay parallelism was evaluated via F-test to compare slope of the serially diluted toenail extract to the slope of the binding curve (percent bound vs. log[concentration]) of the hormone standards. Assay accuracy was evaluated via graphing apparent concentration vs. standard concentration and inspecting goodness of fit of the linear regression line (*r*^2^ > 0.95) and assessing whether the slope was between 0.7 and 1.3 (ideal slope = 1.0).

Baseline hormone concentrations for each elephant were estimated via an iterative process by removing hormone data points that were >2 SD) above the mean, until none of the remaining samples exceeded this limit. The average of remaining samples is considered to represent ‘baseline’ for that hormone in that elephant. Any samples more than two times this baseline was termed ‘elevated’, and any samples also exceeding 2 SD above this baseline was termed a probable ‘peak’, i.e. with ‘peaks’ conceptualized as unusually high elevations. Hormone data are continuous in nature, and physiologically relevant peaks are not always easily identifiable; our thresholds for ‘elevated’ and ‘peak’ were intended for initial exploratory assessment of toenail hormone profiles but are not intended as a definitive determination of physiological relevance. Average concentration of hormones between species were compared using Welch’s *t*-test using the hormone concentrations of all samples from each individual. Pearson correlations were measured between the hormone concentrations within samples from the same individuals. Hormone concentrations were log-transformed before these statistical tests, due to non-normal distribution.

Lastly, hormone and growth rate data were combined to assign an estimated date to each hormone data point. The estimated date was determined by dividing the length of the toenail (in mm) by the individual’s average growth rate to yield an approximate number of days since growth (i.e. since emergence from the nail bed). The number of days was then subtracted by the date of toenail collection to yield an estimated growth date. Hormone ‘peaks’ and estimated growth date were compared to known physiological data for biological validations.

Averages and Welch’s *t*-tests were calculated using Microsoft Excel version 2309. F-test parallelism and Pearson correlation tests were conducted using Graphpad Prism version 9.3.1 for Windows (www.graphpad.com). Individual growth rates and hormone results are expressed as mean ± SD (mean ± SEM for species growth rates and Elephantidae growth rate). The significance threshold was set at alpha = 0.05.

## Results

### Growth rate

The average toenail growth rate for African and Asian elephants combined was 0.21 ± 0.030 mm/day (mean ± SEM). For African elephants, the average growth rate was 0.18 ± 0.015 mm/day with variation among individuals ranging from 0.15 to 0.20 mm/day (Table 2)**.** For Asian elephants, the average growth rate was 0.24 ± 0.034 mm/day with variation ranging from 0.18 to 0.33 mm/day (Table 2). Asian elephant toenail growth rates tended to be faster than African elephants, but this difference was not significantly different (*P* = 0.1825, *n* = 3 African elephants, *n* = 4 Asian elephants; *t* = −1.6109, df = 4, Welch’s *t*-test). Five of seven elephants exhibited higher growth rate in warmer months (March–September), though low sample size and the 1-year study duration precluded formal statistical evaluation of potential seasonal changes in growth rate.

**Table 2:** Toenail growth rate results from African elephants^a^ (*n* = 3) and Asian elephants^b^ (*n* = 4). Average growth rate was calculated as the grand average of the biweekly growth rate and the total growth rate

**Elephant**/**Species**	**Biweekly growth rate (mm/day)**	**Total growth rate (mm/day)**	**Average growth rate (mm/day)**
Anna^a^	0.15 ± 0.09	0.15	0.15
Felix^a^	0.17 ± 0.10	0.18	0.18
Samson^a^	0.20 ± 0.09	0.20	0.20
***African elephant average***			**0.18**
Kamala^b^	0.22 ± 0.13	0.19	0.20
Maharani^b^	0.18 ± 0.10	0.17	0.18
Spike^b^	0.36 ± 0.24	0.31	0.33
Swarna^b^	0.24 ± 0.18	0.22	0.23
***Asian elephant average***			**0.24**

### Assay validations

**Fig. 2:**
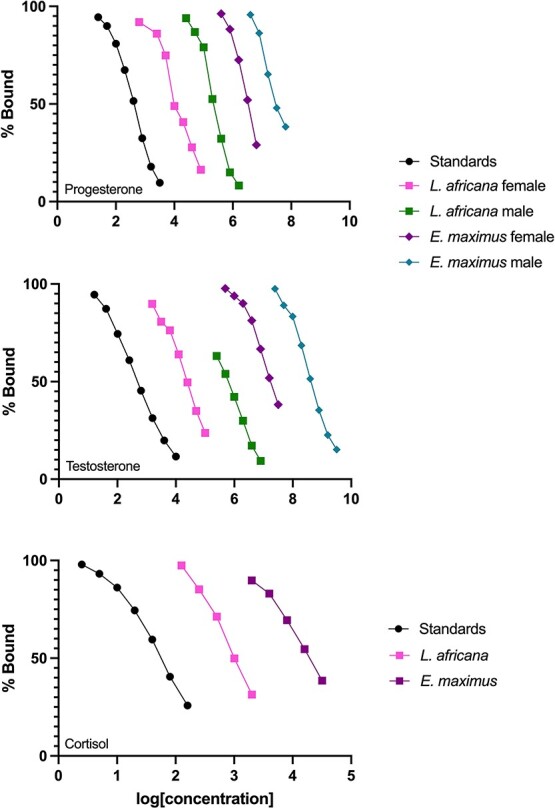
Parallelism results for enzyme immunoassays tested with serially diluted pools of elephant toenail extract for progesterone (top), testosterone (middle) and cortisol (bottom).

Progesterone and cortisol assays demonstrated good parallelism for toenail extracts of both species (Table S2 in Supplementary Information). The testosterone assay, however, demonstrated parallelism for African elephant males but non-parallelism in African elephant females and both sexes of Asian elephants (Table S2 in Supplementary Information). The slopes of the central portions of the curves, however, were generally similar (Fig. 2, middle panel), interpreted here to indicate presence of a probable androgen that binds relatively well to the testosterone assay antibody. Finally, all assays passed accuracy testing, with observed vs expected dose curves being linear and possessing a slope within a desired range of 0.7–1.3 (Table S3 in Supplementary Information).

### Hormone concentrations

Our final sample size was *n* = 83 toenail trimmings, all of which had quantifiable concentrations of immunoreactive progesterone, testosterone and cortisol. One sample was found to be considerably lower in hormone than the rest of the samples but was not omitted from the data set. Across all elephants, toenail progesterone content ranged from 0.5 to 588 ng/g, testosterone ranged from 8 to 2930 ng/g, and cortisol ranged from 2 to 116 ng/g. Average concentrations of all three hormones were higher for African elephant toenail samples than in Asian elephant toenail samples (progesterone: Africans, 139 ± 139 ng/g; Asians, 54 ± 18 ng/g; testosterone: Africans, 404 ± 745 ng/g; Asians, 144 ± 306 ng/g; cortisol: Africans, 32 ± 25 ng/g; Asians, 12 ± 5 ng/g). These species differences were significant for progesterone, testosterone and cortisol concentrations (all *P* < 0.0001, *n* = 29 samples for the African elephants, *n* = 54 for the Asian elephants; progesterone: *t* = 4.5404; df = 55. Testosterone: *t* = 4.5077; df = 62. Cortisol: *t* = 5.8709, df = 44, Welch’s *t*-test). Female African elephants had significantly higher average toenail progesterone (120 ng/g) than Asian elephant females (51 ng/g; *P* = 0.0004, *n* = 19 samples from the African females, *n* = 39 samples from the Asian females; *t* = 3.8571, df = 43, Welch’s *t*-test). Average toenail testosterone was higher for the African male (890 ng/g) than the Asian male (328 ng/g), but this difference was not significantly different (*P* = 0.1319, *n* = 10 samples from the African male, *n* = 15 samples from the Asian male; *t* = 1.5784, df = 18, Welch’s *t*-test).

**Fig. 3:**
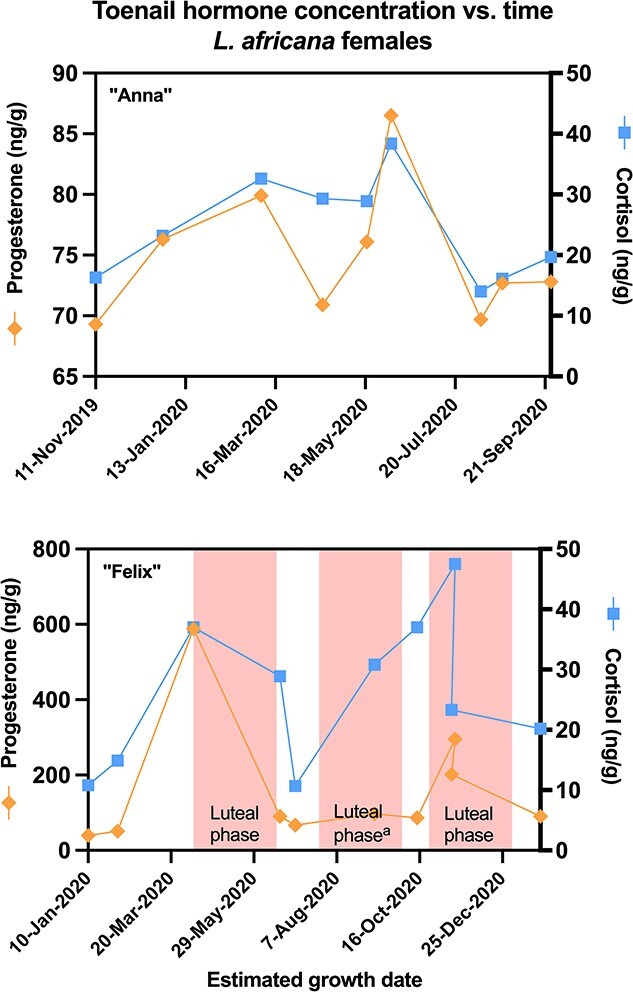
Concentration of progesterone and cortisol graphed against estimated toenail growth date for female African elephants (*L. africana*) Anna (top) and Felix (bottom). Sampled toenails of African elephants reflected expected acyclicity (Anna) and historical ovarian cycling (Felix). Coloured bars indicate independently confirmed luteal phases. ^a^This luteal phase was classed as ‘abnormal’ by zoo staff.

**Fig. 4:**
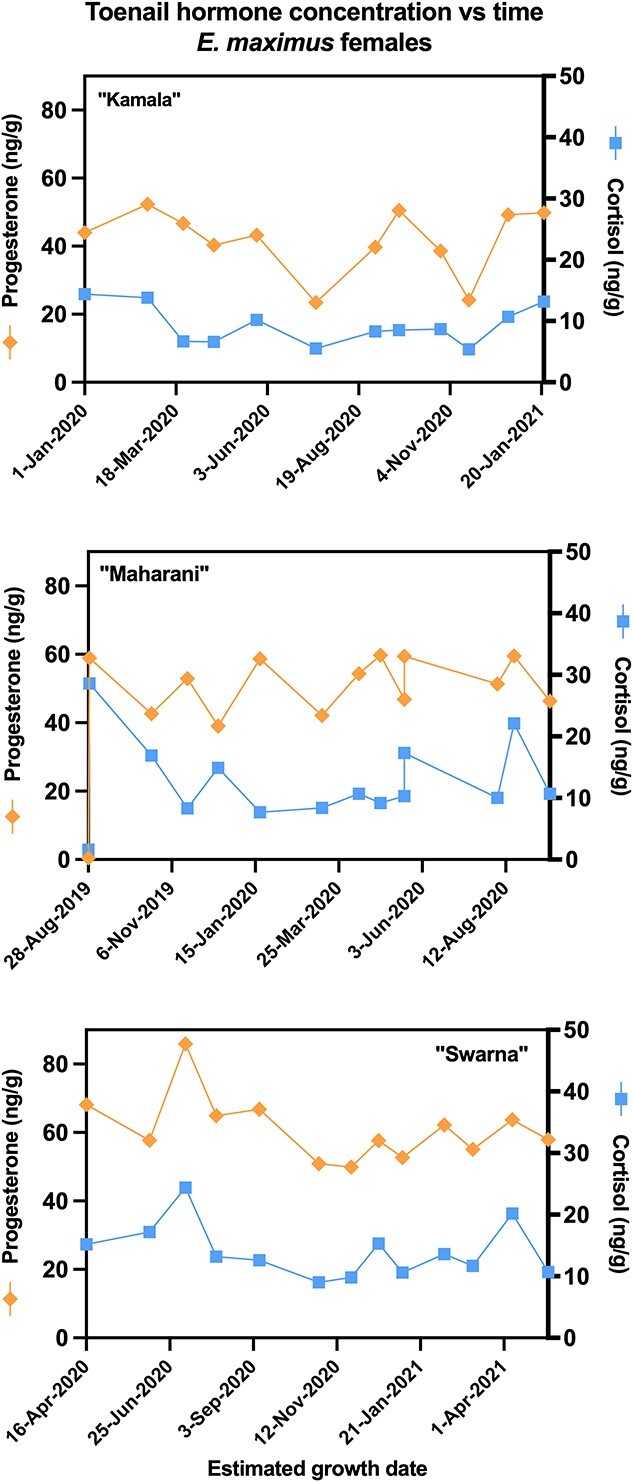
Concentration of progesterone and cortisol graphed against estimated toenail growth date for female Asian elephants (*E. maximus*) Kamala, Maharani and Swarna. Asian elephant toenails did not show evidence of present or historical ovarian cycling, with minimal fluctuation between samples.

**Fig. 5:**
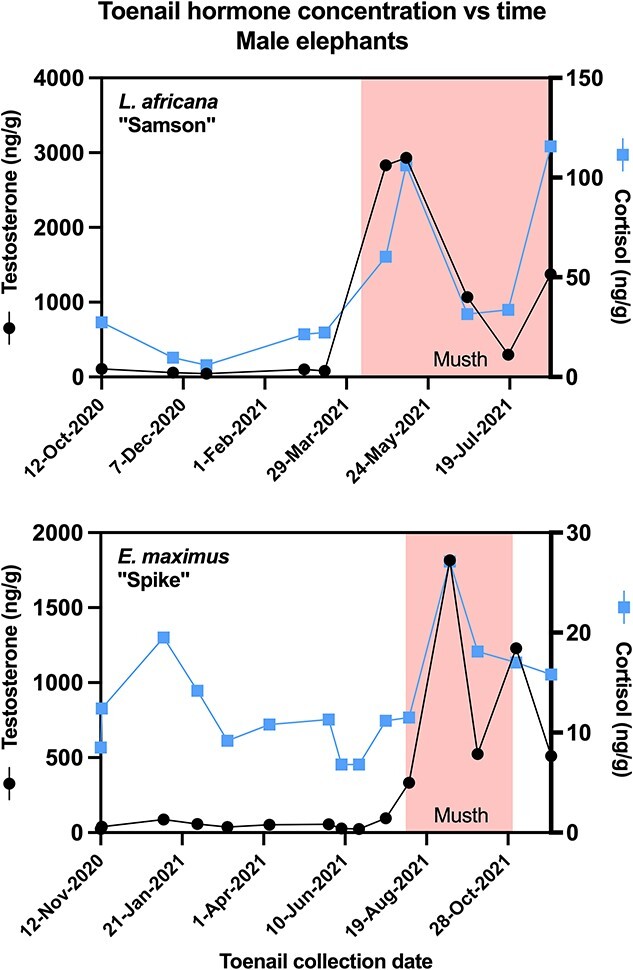
Concentration of testosterone and cortisol graphed against the toenail collection date for two male elephants, African elephant (*L. africana*) Samson (top) and Asian elephant (*E. maximus*) Spike (bottom). Toenail samples containing high testosterone were collected while individuals were actively in musth (coloured bars).

Longitudinal hormone profiles for each individual elephant, with estimated dates of growth at the nail bed, are presented in Figs 3 and 4. With a few exceptions (Table 3), samples with high reproductive hormone concentrations also tended to be high in cortisol (*P* < 0.05). For the two acyclic females, testosterone and cortisol were not correlated (Figs 2 and 3; Table 3). One of the three cycling females, African female ‘Felix’, displayed high variation in hormones, with higher concentrations than other females, and estimated growth dates of peak samples corresponding with her known ovarian cycles (Fig. 3). The remaining females all had similar toenail hormone concentrations (range: 0.5–90 ng/g) with fluctuations that did not resemble apparent ovarian cycling (Figs 3 and 4). Some toenail samples from the two males had high testosterone concentrations (Fig. 5), but estimated growth dates did not correspond with prior musth cycles. Rather, for both these males, the high-testosterone toenail samples were collected during a current musth episode (i.e. the individual was in musth when that toenail sample was collected). Additionally, when an elephant was in musth, toenail trimmings collected from female elephants at the same zoo also showed minor elevations in testosterone (see Fig. S1 in Supplementary Information).

**Table 3:** Pearson correlation statistics of log-transformed hormone concentrations within each elephant. Significance is denoted via superscripts.

**Elephant**	**Progesterone–testosterone**	**Progesterone–cortisol**	**Testosterone–cortisol**
Anna	0.3331	0.8056^*^	−0.1754
Felix	0.7771^*^	0.7228^**^	0.8548^*^
Samson	0.9479^***^	0.8719^***^	0.8817^***^
Kamala	0.4254	0.7411^*^	0.7062^**^
Maharani	0.4707	0.8193^***^	0.7304^*^
Spike	0.7768^***^	0.6735^*^	0.8103^***^
Swarna	0.1408	0.7985^*^	0.3022

^*^ *P* < 0.01;

^**^ *P* < 0.05;

^***^ *P* < 0.001.

## Discussion

Toenails of African and Asian elephants contained detectable and quantifiable concentrations of progesterone, testosterone and cortisol when analyzed with commercial EIA kits. All assays passed validation tests of parallelism and accuracy for toenail extract of both elephant species (albeit with one case of minor non-parallelism), indicating presence of likely hormones in toenail extract (i.e. extract contains substances that bind well to the assay antibodies) as well as good mathematical accuracy of the assay across a range of concentrations. Prior studies have demonstrated that claws of some other vertebrates, and fingernails of humans, also contain detectable steroid hormones. To our knowledge, our study is the first to test elephant toenails for steroid hormone content. In combination with prior studies, our results suggest that claws and nails of many other vertebrates, as well as potentially hoof of artiodactyls and perissodactyls, may also contain steroid hormones.

The temporal period represented by any keratin sample type requires careful consideration, as there can be a substantial lag in time between deposition of hormone in the growth zone and emergence of (or collection of) the sample distally at a later time point. Though there have been at least three prior efforts to estimate the toenail growth rate in elephants (Seilkopf, 1959; Benz, 2005; Fowler and Mikota, 2006), none of those reports have been peer-reviewed. Our study included a year-long effort at evaluating typical toenail growth rate for multiple individuals and both sexes of two elephant species. Based on our estimated average growth rate for Elephantidae of 0.21 mm/day, a large toenail could contain over a year’s worth of hormone concentrations deposited across the nail from proximal cuticle down to the distal tip. Growth rate ranges of 0.17–0.33 mm/day reported by Fowler and Mikota (2006) were similar to our observed ranges: 0.15–0.33 mm/day. Our estimated growth rates are also similar to those of Benz (2005; unpublished thesis) for African (5.4 mm/28 days) and Asian (7.0 mm/28 days) elephants. Benz (2005) additionally found that Asian elephants had higher toenail growth rate compared to Africans. We also observed growth rate variation between species and individuals and some indication of seasonal differences (data not shown). However, additional samples, individuals and years would be necessary to determine if these differences truly reflect variation by sex, season or time of year.

It is possible that substrate and locomotory patterns may affect toenail wear and growth rate. Substrate and locomotion may vary seasonally, as elephants in North American zoos often spend more time within barns in winter than in summer. The anatomy of the elephant foot is complex and is believed to house a pressure mechanism that increases circulation to the foot when actively walking (Fowler and Mikota, 2006); it is possible, therefore, that locomotion may affect circulation to the nail bed and hence may directly affect toenail growth (i.e. through mechanisms other than increased nail wear). These questions await further study. It may be fruitful to combine studies of locomotion and growth rate with studies of clinical care, as locomotion is an important element in foot health in elephants (Fowler and Mikota, 2006).

Biweekly growth rates were evaluated in order to inspect potential variation in nail growth rate during the observation period, but were predicted to have greater measurement error, while the total growth rate was considered to have less measurement error (due to the greater length of toenail being measured), but measurement error does occur even with total growth rates. Due to the difficulties inherent in measuring distances with ~1 mm precision on a living, unrestrained, elephant’s foot, some measurement error is inevitable with both methods. We therefore considered the most accurate estimate of growth rate to be the combined average of both of these methods.

Concentrations of hormones in toenails showed variation between samples, individuals, sexes and species. Cortisol concentration tended to correlate with testosterone and progesterone, with only a few cortisol elevations that did not match reproductive hormone elevations. This notable correlation among all steroid hormones is a common finding in all vertebrate sample types and is thought to be due in part to the fact that progesterone is the precursor to all the steroids, and in part because reproduction is itself a type of stressor. For example, cortisol and androgens typically show positive correlations across musth cycles in males (e.g. LaDue *et al.,* 2023), presumably to help animals cope with the energetic burden of reproductive behavior and reproductive physiology. In female elephants, diverse positive, negative and no correlations have been noted between glucocorticoids and the reproductive steroids, varying with the nature of the reproductive event (e.g. Bechert *et al.* 1999; Brown *et al.,* 2004; Oliveira *et al.,* 2008; Fanson *et al.,* 2014; Kajaysri and Nokkaew, 2014; Glaeser *et al.,* 2020; Towiboon *et al.,* 2022). Generally, it is believed that positive correlations between glucocorticoids and the reproductive hormones can occur during normal reproductive cycling in vertebrates, while negative correlations are more likely to indicate occurrence of unpredictable, non-reproductive or unusually severe stressors (reviewed in Romero & Wingfield 2016). Overall, in this study, cortisol patterns did not suggest occurrence of any unusual stressful events during the study period or the prior year. We hypothesize, though, that acute stress (short-term stress) may not be reflected in toenail endocrine data, due to the short period in which glucocorticoids would be deposited into the toenail. It is also possible that the strong correlations observed here across hormones may represent variations in deposition rate of steroids into toenail matrix that may not necessarily reflect circulating concentrations. Parallel studies of plasma and toenails collected from the same elephants across two or more years could shed further light on this issue. Such studies should be possible, since many elephants in human care are habituated to routine blood collection as well as routine foot care.

The reproductive hormones progesterone and testosterone showed variable patterns. Testosterone was elevated in toenail samples trimmed while the male was actively in musth, rather than reflecting prior occurrence of a historical musth cycle. This finding suggests that even after an increment of toenail is grown and emerges from the nailbed, it can still acquire hormone through some as-yet-undetermined route. Male elephants in musth routinely dribble urine onto their legs (urine dribbling is one of the diagnostic indicators of musth), and urine is one of the excretion routes for testosterone, and thus urine may have deposited testosterone onto the external nail surface. While it would be expected that urine dribbling would deposit testosterone primarily onto hind feet, increased testosterone was observed in toenails from both front and hind feet. Alternatively, hormones could be entering nails simply due to the elephant walking through areas of substrate containing urine or faeces (i.e. affecting both front and hind feet), or via some other route, such as skin oil or from the underlying nail bed (Palmeri *et al.,* 2000). Intriguingly, when a male was in musth at a given zoo, toenail trimmings collected from nearby females from the same zoo also had minor elevations in testosterone. It is not clear whether this simply represents widespread contamination of all the elephants’ feet and toenails, or whether other elephants might react physiologically to the nearby presence of a musth male.

In contrast to testosterone data, progesterone data of at least one female corresponded to prior ovarian cycles and not current ovarian cycles. Toenails believed to have grown during two of African elephant Felix’s luteal phases showed elevated progesterone. Thus, toenail progesterone content may indicate whether a female experienced a luteal phase in the prior year. However, one Felix toenail believed to have grown during a luteal phase did not show elevated progesterone, though this particular luteal phase had been noted by keepers as “abnormal” (Fig. 3, superscript a). Felix was the only one of three cycling females that showed clear evidence of such a pattern (i.e. high toenail progesterone corresponding to a documented prior ovarian cycle). The other two cycling females, and both of the acyclic/irregular females, had much lower progesterone with erratic but not notable elevations. Therefore, further study will be necessary to verify whether toenail progesterone is a reliable indicator of female ovarian cycling in the prior year. The slow growth rate of toenail may mean that toenail endocrine data are more suitable for determination of the very long progesterone elevations of pregnancy, rather than the briefer elevations of luteal phases. Follow-up studies examining toenail progesterone patterns across full pregnancies would be fruitful.

Additional research is needed to further evaluate the utility of monitoring hormone concentration in toenails for elephant conservation. For future studies, we recommend sampling the largest toenail on a foot; comparing front to back feet (especially for males, across musth cycles); regularly measuring the whole length of the toenail; comparing freeze-drying to air-drying nails; and evaluating additional hormones (e.g. estradiol, thyroid hormones). Possible effects of season or locomotion on toenail growth also should be considered. Studies of more individuals, ideally including multiple cycling females as well as musth males, will enable better assessment of whether reproductive status of the prior year is consistently reflected in toenail endocrine data. Such studies could be paired with ongoing plasma collections and accelerometer locomotion studies.

New tools and techniques for wildlife endocrinology could allow for the enhancement of methodologies and research of *in situ* and *ex situ* elephants. Examination of hormone patterns in toenails could allow conservationists to look into the past at physiological profiles of elephants both living and deceased. Toenails are regularly trimmed as a part of regular care for *ex situ* individuals, and the trimmings can be readily collected and can be stored indefinitely at room temperature once dried. Thus, toenail samples could function as a long-term hormone bank for conservation programs to retrospectively evaluate individual physiology. Toenails collected from deceased individuals in captivity, the wild or in archived museum samples can be trimmed into successive pieces to reconstruct a hormone profile and physiological history leading up to the individual’s death. It is possible, too, that wild elephants tranquilized for other procedures could be sampled by means of collecting small samples from the toenail surface from top to bottom to assemble hormone histories. Other analyses commonly employed on keratin samples (e.g. drug analysis, stable isotopes, mineral status; Palmeri *et al.,* 2000; Trueman *et al.,* 2019; Sach *et al.,* 2020) may allow for insight into relationships among physiological state, diet and other relevant biological information. Continued work is needed, but elephant toenails could provide conservationists with a new tool to monitor individual physiology.

## Supplementary Material

Web_Material_coae048

## Data Availability

Data utilized for this article can be accessed by requesting access from the corresponding author.
