# Supplementary material for: Nailing it: Investigation of elephant toenails for retrospective analysis of adrenal and reproductive hormones
Source: Conserv Physiol. 2024 Aug 2;12(1):coae048. doi: 10.1093/conphys/coae048 (PMC11295213; doi:10.1093/conphys/coae048)
Supplement: Web_Material_coae048 [file web_material_coae048.pdf]

Supplementary Information

Table S1. Intra-assay and inter-assay coefficients of variation (%CV), based on interpolated hormone concentrations of African elephant and Asian elephant toenail extract pools. Intra-assay %CV is based on six replicates assayed in the same assay; inter-assay %CV is based on six replicates assayed in six different assays.

Species	Hormone	Intra-assay %CV	Inter-assay %CV
African elephant	Progesterone	3.33%	3.57%
	Testosterone	2.43%	3.13%
	Cortisol	3.12%	4.81%
Asian elephant	Progesterone	3.25%	1.42%
	Testosterone	0.83%	2.07%
	Cortisol[a]	3.77%	6.84%

[a]These assays utilized a 1:5 dilution of antibody and conjugate. See methods section for more information on this technique.

Table S2. Parallelism statistics for enzyme immunoassays for three steroid hormones in toenail extract of African and Asian elephants. Parallelism was assessed using an F-test, with a significance threshold (alpha) of 0.05.

Species	Hormone	Sex	Standard curve slope (log[conc]/%Bound)	Sample pool slope (log[conc]/%Bound)	P-value (<0.05)
African elephant	Progesterone	Female	-43.90	-45.99	0.7477
		Male	-60.86	-70.62	0.1653
	Testosterone	Female	-34.33	-41.32	0.0198
		Male	-34.66	-37.52	0.1197
	Cortisol	Female/Male	-53.92	-60.82	0.1148
Asian elephant	Progesterone	Female	-64.03	-65.88	0.6637
		Male	-64.03	-53.51	0.1626
	Testosterone	Female	-37.66	-47.89	<0.0001
		Male	-37.66	-51.39	<0.0001
	Cortisol	Female/Male	-52.70	-49.21	0.0604

Table S3. Results of assay accuracy testing for three hormones in toenail extract of African and Asian elephants. Slope was calculated from the line of apparent dose vs. relative dose of standards spiked with pooled extract, with apparent dose derived from comparison to unspiked standards. A slope within 0.7-1.3 and an $r^2 > 0.95$ (best fit linear regression line) indicates acceptable accuracy.

Accuracy pool	Hormone pool	Dilution	Slope (0.7-1.3)	R^2 (>0.95)
African elephant	Progesterone	1:4	0.8326	0.9996
	Testosterone	1:51	1.0650	0.9967
	Cortisol	1:4	1.0520	0.9956
Asian elephant	Progesterone	1:4	0.8219	0.9989
	Testosterone	1:4	1.1600	0.9982
	Cortisol	1:1	0.9760	0.9996

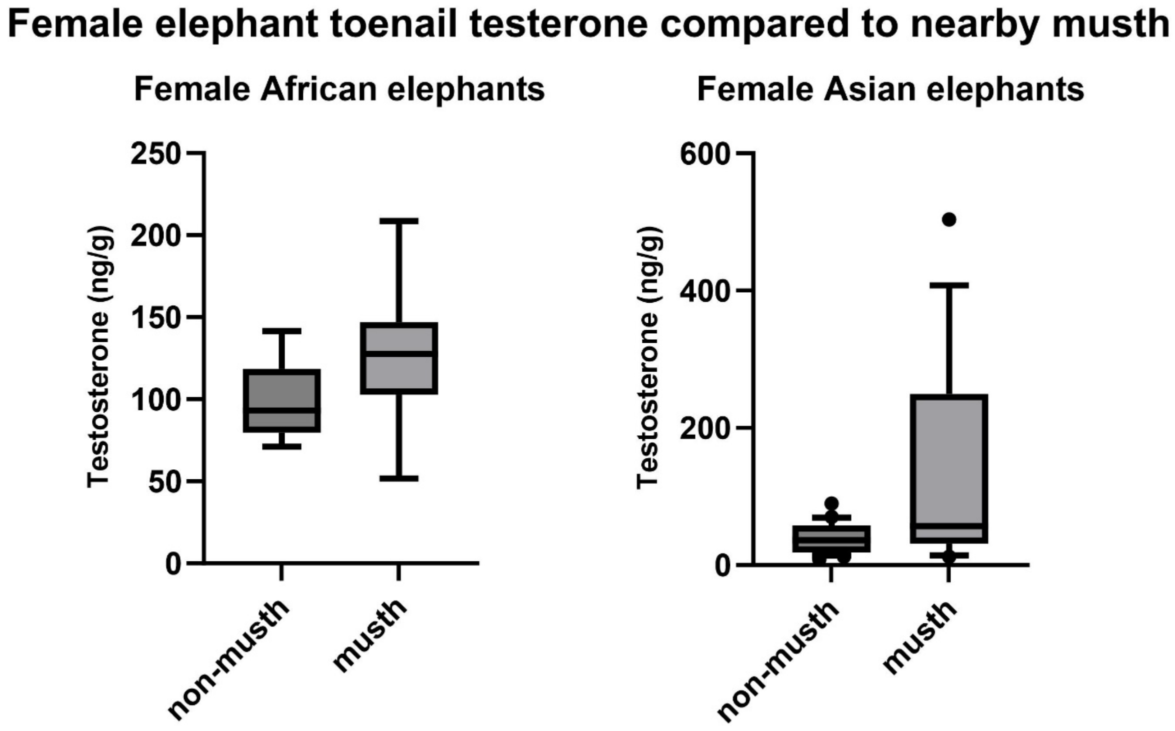

Figure S1. Concentrations of testosterone in collected female toenails when a male elephant housed as the same facility was in musth at the time of collection, compared to non-musth toenail samples. (Samples from known luteal phases for cycling female African elephant Felix were excluded, due to the documented positive correlation between progesterone and testosterone in female luteal phase samples.)
